# Give and Take

**Published:** 1891-02-28

**Authors:** 


### Passing Topics.

### GIVE AND TAKE.

"Do you know," asked Lord Sandhurst of Dr. Ord, of St. Thomas's, "if you collect patients from the country?" "Yes," answered the doctor, "a good many cases come up from the country." "Seeking," pursued his Lordship, "rather the advice of a great London Hospital with a great reputation, than that which they can obtain in their own country town?" "Not only that," replied Dr. Ord, "but a good many country doctors send up their difficult cases, using the hospital for consultation as it were." "Is there any charge made by the hospital for these cases?" "No." "Or in the case of a patient coming up from the country and taking up a bed in the hospital, is there any charge made upon the locality?" "No."

Thus much for country; now for town. The witness is Mr. Todd, the secretary of St. George's. "Are you well supported by residents in the neighbourhood?" inquires the chairman. "We are not nearly so well supported as we could expect from our position; a good many houses do not give us any subscriptions." "And at the same time, perhaps, they send their servants to the hospital?" "Yes; we have a good many servants whose masters or mistresses we know are not subscribers."

We have quoted this at length because we have here presented in a concrete form evidence of that want of sympathy —one might almost say of common honour—which a large number of well-to-do people are prone to exhibit in their dealings with our hospitals. It has been the fashion, and not without reason, to attribute to the working classes a callous indifference to the necessities of the institutions which confer so vast benefits upon their order. Reprehensible enough is the selfish unconcern of those well-paid artizans who year by year resort to the hospitals without making the feeblest effort to assist towards their maintenance, and claim for themselves and for every member of their families all needful medical relief without being at the pains even to consider how its cost is met. Their offence is, however, venial indeed side by side with that of the rich ladies who scour the town for "letters" with which to procure admittance for their dependents, or with the same object in view, turn to account a chance acquaintance with a member of the visiting staff, and who, when success has attended their efforts, accept unblushingly from grateful friends the credit of a generous and philanthropic act. There are hospitals where the stoppage of a carriage is always looked upon as symptomatic of favours to be asked or, rather, of demands to be made, and the extent of the requisition is, it is said, in the exact ratio of the magnificence of the equipage. Thus a one-horse brougham may mean nothing more than a request for a list of subscribers and a faltering refusal to contribute, while a carriage and pair with two powdered servants usually portends a demand for immediate admission and a haughty resentment of any reference to rules.

Let the Lords' Committee rest assured the hospitals see nothing of reciprocity, whether on the part of individuals or localities. To admit the obligation implied in the suggestion is the last thing thought of. Employers, patrons, kinsmen, and the general practitioners who want another opinion, or whose fees are no longer safe, all send up their cases without compunction, and it is no concern of theirs how the hospital is kept going. The Lords' Committee will have accomplished a great thing for hospitals when they have taught the public their duty in this respect. A great deal of the financial difficulty suffered by some of the most useful of our hospitals arises from the fact that exactly ninety-nine out of a hundred people of all classes are prepared to take what they can get and to give back nothing in return. Agricola.

